# Case report and literature review: management of Paxlovid (nirmatrelvir/ritonavir)-induced acute tacrolimus toxicity in a patient with systemic lupus erythematosus

**DOI:** 10.3389/fphar.2024.1364121

**Published:** 2024-06-19

**Authors:** Chenxiao Jiang, Xiaodi Yan, Peng Xia, Xuemei Luo, Haoyue Zheng, Hanwen Tong, Yun Liu, Huaijun Zhu, Peng Xu, Jun Wang

**Affiliations:** ^1^ Department of Pharmacy, Nanjing Drum Tower Hospital, Affiliated Hospital of Medical School, Nanjing University, Nanjing, Jiangsu, China; ^2^ School of Basic Medicine and Clinical Pharmacy, China Pharmaceutical University, Nanjing, Jiangsu, China; ^3^ Department of Pharmacy, Nanjing Medical University, Nanjing, Jiangsu, China; ^4^ Women’s Hospital of Nanjing Medical University, Nanjing Women and Children’s Healthcare Hospital, Nanjing, China; ^5^ Department of Emergency Medicine, Nanjing Drum Tower Hospital, Affiliated Hospital of Medical School, Nanjing University, Nanjing, Jiangsu, China

**Keywords:** systemic lupus erythematosus, tacrolimus, nirmatrelvir/ritonavir, CYP induction, case report

## Abstract

Despite the availability of effective vaccines and treatments for SARS-CoV-2, managing COVID-19 in patients with systemic lupus erythematosus (SLE) remains challenging, particularly considering drug-drug interactions (DDIs). Here, we present a case of DDIs between Tacrolimus (Tac) and nirmatrelvir/ritonavir (NMV/r) in a 32-year-old male with SLE. Following self-administration of NMV/r and resumption of Tac after 5 days, the patient experienced acute nephrotoxicity and neurotoxicity, accompanied by supratherapeutic Tac levels, despite Tac being withheld during NMV/r. The primary cause of this acute toxicity is attributed to ritonavir’s inhibitory effect on both CYP3A4 enzymes and P-glycoprotein. Upon admission, Tac was discontinued, and supportive therapies were initiated. Phenytoin, a CYP3A4 inducer, was administered to lower Tac levels under the guidance of clinical pharmacists, effectively alleviating the patient’s acute toxic symptoms. The half-life of Tac during the treatment of phenytoin was calculated to be 55.87 h. And no adverse reactions to phenytoin were observed. This case underscores the persistence of enzyme inhibition effects and demonstrates the effectiveness and safety of utilizing CYP3A4 enzyme inducers to mitigate Tac concentrations. Furthermore, it emphasizes the importance of healthcare providers and patients being vigilant about DDIs in Tac recipients. Lastly, it highlights the indispensable role of pharmacist involvement in clinical decision-making and close monitoring in complex clinical scenarios. Although our findings are based on a single case, they align with current knowledge and suggest the potential of individualized combination therapy in managing challenging COVID-19 cases in immunocompromised patients.

## 1 Introduction

The COVID-19 pandemic, caused by the novel severe acute respiratory syndrome coronavirus 2 (SARS-CoV-2), has had a profound global impact. In response, the Federal Drug Administration (FDA) granted emergency use authorization (EUA) to nirmatrelvir/ritonavir (Paxlovid, NMV/r) to mitigate the significant morbidity and mortality associated with COVID-19. Systemic lupus erythematosus (SLE), an autoimmune disorder affecting various body systems, is typically managed with calcineurin inhibitors (CNIs), such as Tacrolimus (Tac, or FK506), to suppress abnormal immune responses (Fanouriakis et al., 2019). NMV/r is advocated for treating COVID-19 in SLE patients, who are particularly susceptible to severe complications post-infection (Murphy, 2022). A critical consideration is the pronounced Tac toxicity arising from the ritonavir-Tac interaction.

This case report addresses the reversal of Tac toxicity induced by concurrent NMV/r use through the activation of cytochrome P450 (CYP) 3A4 with phenytoin in an SLE patient. To our knowledge, this is the first reported case of managing phenytoin to mitigate acute Tac toxicity induced by NMV/r in an SLE patient. This case stands out for its novel identification of ritonavir-induced Tac toxicity and is among the few reported instances demonstrating phenytoin’s efficacy and safety in resolving acute Tac toxicity.

## 2 Methods

### 2.1 Case report

The patient data required for the case report were retrieved from the electronic patient information system of Nanjing Drum Tower Hospital. Patient consent for publication was obtained.

### 2.2 Structured review

We searched MEDLINE literature of PubMed by using the search term “Tacrolimus” AND “Nirmatrelvir/ritonavir OR Paxlovid” to identify the relevant reports up to 11 May 2024. Additionally, other databases including Web of Science, Cochrane Database, and ClinicalTrials.gov were searched using the same search term. All the reports obtained were screened for exclusion in the review according to the following criteria: 1) comment, correspondence, and response; 2) meeting abstract, editorial material; 3) unrelated to topic; 4) insufficient information on therapy. After a thorough analysis of the full-text articles, a secondary search was performed to exclude the guidelines, dosing suggestions reviews, data from the Fears database, case reports or case series without acute toxicities, and retrospective studies.

The extracted data included reported country and year, patient demographics, primary indications for Tac, previous Tac and NMV/r doses, NMV/r duration, Tac dose adjustment during NMV/r administration, toxic manifestation, initial maximum Tac level after NMV/r administration, CYP inducers and other treatments, restart Tac doses after treatment, Tac levels after Tac resumption, and patient prognosis.

## 3 Case presentation

### 3.1 Case report

A 32-year-old male, weighing 70 kg, and with a 6-year history of SLE, was admitted to the hospital on 20 August 2023. He had been receiving a long-term regimen of oral Tac 1 mg twice daily and prednisone 5 mg daily. Over the past 3 years, his baseline serum creatinine levels had stabilized at 0.74–0.94 mg/dL, while his Tac blood levels remain untested. Notably, he had never been vaccinated against COVID-19, and his parents had no history of immune-related diseases.

Approximately 2 weeks prior to admission, he presented with symptoms including a fever of 39°C, muscle aches, cough with sputum production, shortness of breath, a widespread rash, diarrhea, and a positive nucleic acid test for COVID-19. He initiated with NMV/r (300 mg/100 mg twice daily) himself 8 days before admission (on August 12), during which his Tac and prednisone were withheld. After 5 days of NMV/r treatment, he noticed improvement in COVID-19 symptoms and resumed his Tac and prednisone. However, the next day, he experienced chest tightness, shortness of breath, and profusely sweating. On August 20, his condition deteriorated, marked by aggravated chest tightness, breathlessness, brownish sputum, muscle pain, nervousness, disorganized speech, involuntary muscle spasms, and tremors, prompting his admission to our hospital’s emergency department.

Upon admission, his laboratory results showed a creatinine of 3.9 mg/dL, hyponatremia (sodium level at 122.6 mmol/L), and mild hyperkalemia (potassium level at 5.23 mmol/L). Electrocardiography (ECG) indicated sinus tachycardia (157 bpm) with ST-T changes. Remarkably, his Tac concentration was exceptionally high at 57.6 ng/mL. He was managed with tracheal intubation, rehydration, sedation and analgesia, anti-infective treatment, continuous venovenous hemodiafiltration (CVVHDF), and additional supportive therapies.

**FIGURE 1:**
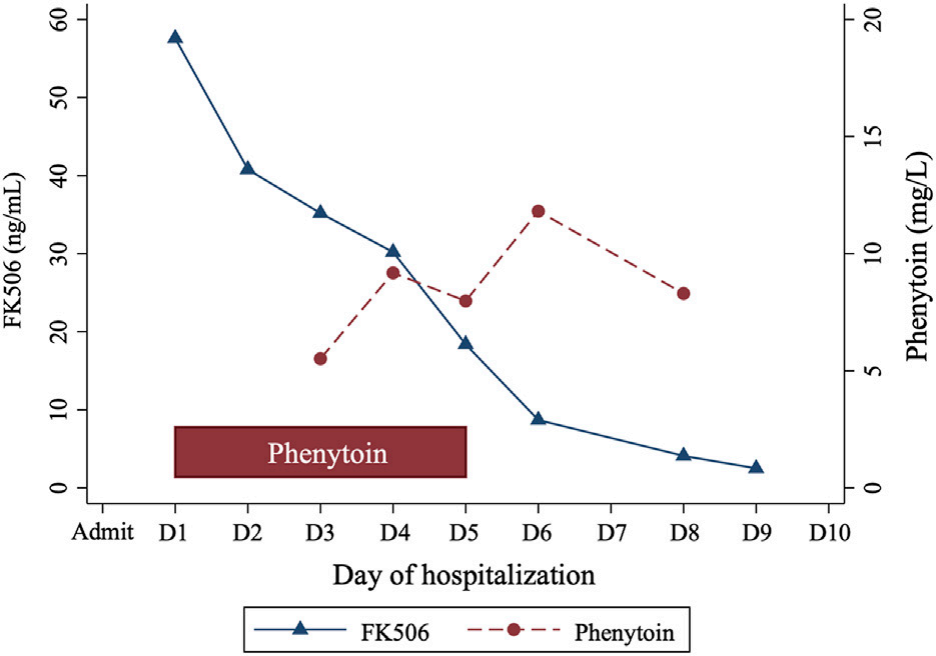
Changes in tacrolimus and phenytoin levels over a 9-day period.

Clinicians sought consultation from the clinical pharmacists on effectively reducing Tac levels. Considering Tac’s high protein binding and limited clearance by continuous renal replacement therapy (CRRT), the pharmacist recommended a cytochrome P450 (CYP) enzyme inducer (either phenytoin or rifampicin) to lower Tac concentrations. The patient received oral phenytoin (100 mg thrice daily) for 5 days, resulting in a gradual decrease in Tac levels from 57.6 ng/mL to 2.5 ng/mL over 8 days (Figure 1). Phenytoin trough level, measured 2 days post-administration, ranged from 5.52 mg/L to 11.82 mg/L. His creatinine levels improved to 1.21 mg/dL. The Tac elimination rate constant (ke) and the half-life (t_1/2_) were calculated as follows (Shiohira et al., 2024):

$$\text{ke}=\text{ln}\left(C1/C2\right)/\left(T2-T1\right)$$

$$t_{1/2}=0.693/\text{ke}$$

The calculated t_1/2_ were days 1–5: 55.87 h (during the treatment with phenytoin), days 6–9: 37.50 h (after treatment with phenytoin).

By the 11th day, the patient regained consciousness with normal cerebrospinal fluid tests. He was extubated on day 17, showing no respiratory symptoms or muscle tremors but experiencing dysphagia. Brain magnetic resonance imaging showed no abnormalities. Upon discharge, his condition had improved significantly, with creatinine at 0.32 mg/dL, sodium at 136.1 mmol/L, and potassium at 3.34 mmol/L. He was discharged without Tac, and prescribed hydroxychloroquine (200 mg twice daily), methylprednisolone (20 mg daily), and potassium chloride (1 g thrice daily). No adverse reactions to phenytoin were observed, and there was no evidence of SLE recurrence or exacerbation. A follow-up after 1 month showed symptomatic improvement, albeit with an increased heart rate, necessitating maintenance therapy with prednisolone (8 mg daily) and metoprolol (47.5 mg daily).

### 3.2 Review

**FIGURE 2:**
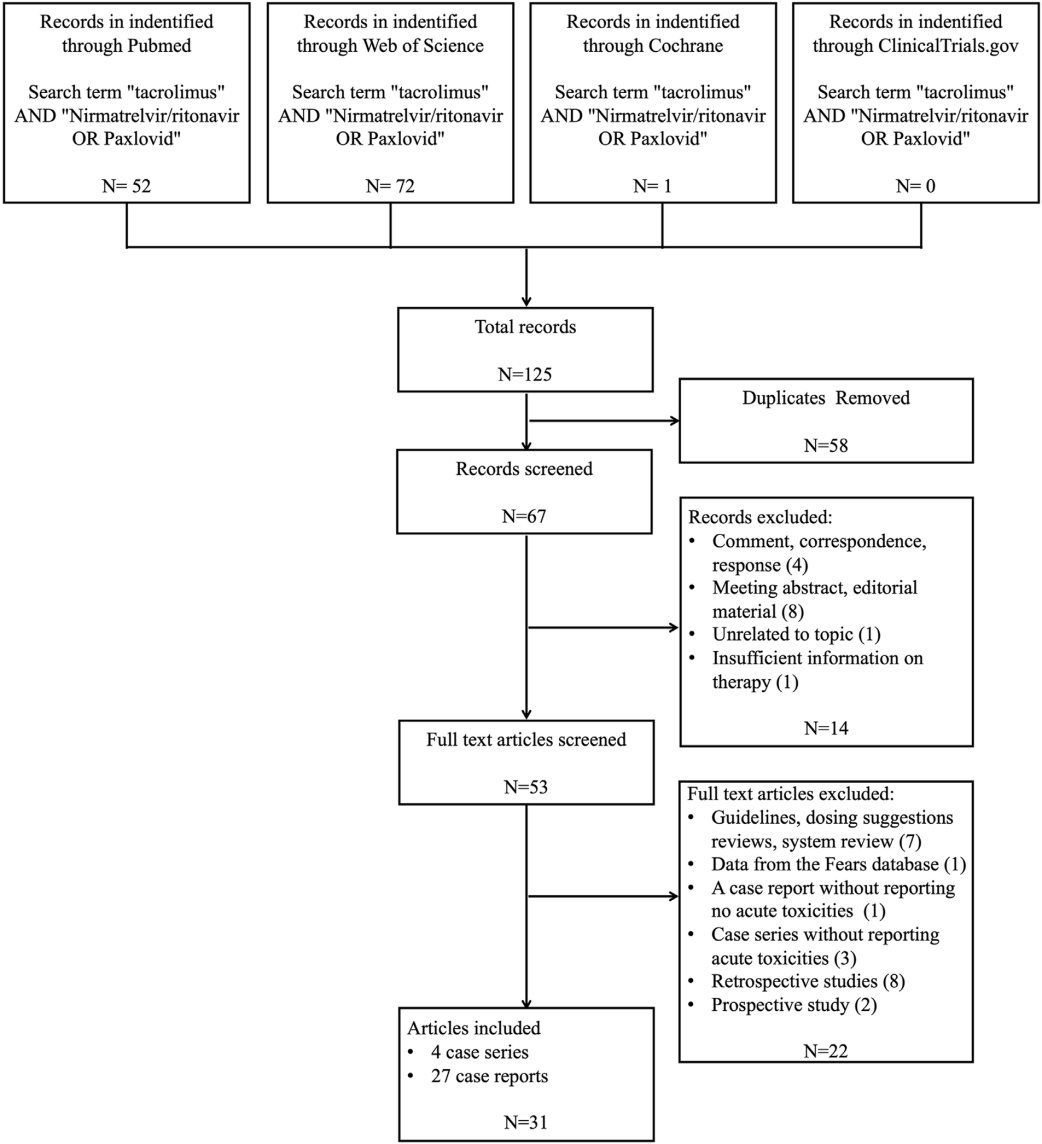
A flowchart of the selection process of included articles in this review.

We encompassed 31 publications (Figure 2), comprising 27 case reports and 4 case series, involving a collective of 42 patients, among whom 38 experienced Tac toxicities. Table 1 summarizes the demographics, clinical manifestations, treatments, and outcomes. Patients’ age ranged from 13 to 85 years, with 21 (50.0%) being male. The primary indications for Tac use included: 36 (85.7%) solid organ transplants, 2 (4.8%) systemic lupus erythematosus, 1 (2.4%) myasthenia gravis, 1 (2.4%) hematopoietic stem cell transplant, 1 (2.4%) ulcerative colitis, and 1 (2.4%) interstitial pneumonia associated with dermatomyositis.

**TABLE 1:** Characteristics, clinical presentation, and outcomes of patients with acute tacrolimus toxicities after Paxlovid administration from case reports and case series.

Country/Year/Ref	Sex	Age	Primary indications for Tac	Previous Tac doses	NMV/r doses	NMV/r duration	Tac dose adjustment during NMV/r administration	Toxic manifestation	Initial maximum Tac level after NMV/r using	CYP inducers treatment	Other treatments	Restart Tac doses after treatment	Tac levels after Tac resumption	Prognosis
USA/2022/(Prikis and Cameron, 2022)	M	34	Kidney transplant	2 mg every 12 h	150/100 mg twice daily	3 days (5 doses)	No adjustment	Nausea, vomiting, acute kidney injury	>30 ng/mL	No	Hold Tac	2 mg every 12 h (when Tac level was 8.8 ng/mL)	4–6 ng/mL	Recovered
Israel/2022/(Berar et al., 2022)	F	23	Kidney transplant	2 mg twice daily	300/100 mg twice daily	1.5 days (3 doses)	Reduce Tac to 1 mg twice daily	Increased serum creatinine	92.4 ng/mL	No	NR	NR	NR	Recovered
USA/2023/(Modi et al., 2023)	M	74	Post-orthotropic heart transplantation	1 mg twice daily	300/100 mg twice daily	5 days	Resume 1 mg twice daily	Headaches, vertigo-like symptoms, tremors, a metallic taste, acute kidney injury	>60 ng/mL	No	Hold Tac	1 mg twice daily (when Tac level was 15.7 ng/mL)	4.7 ng/mL	Recovered
											Intravenous hydration with lactated ringers			
China/2023/(Liu et al., 2023)	F	62	Kidney transplant	2 mg daily	300/100 mg twice daily	5 days	Discontinue Tac	No adverse reactions	11.9 ng/mL	No	NR	2 mg daily (when Tac level was <5 ng/mL)	3.5 ng/mL	Recovered
	M	48	Kidney transplant	2 mg daily	150/100 mg twice daily	5 days	Discontinue Tac	Nausea, vomiting, increased serum creatinine	24.3 ng/mL	No	NR	2 mg daily (when Tac level was <5 ng/mL)	5.1 ng/mL	Recovered
	F	32	Kidney transplant	2 mg daily	300/100 mg twice daily	5 days	Discontinue Tac	Increased serum creatinine	>30 ng/mL	No	NR	2 mg daily (when Tac level was <10 ng/mL)	5.1 ng/mL	Recovered
USA/2022/(Young et al., 2023)	F	14	Kidney transplant	2.5 mg twice daily	300/100 mg twice daily	1 day (2 doses)	No adjustment	Increased serum creatinine	54 ng/mL	No	Hold Tac	2.5 mg AM/2 mg PM (when Tac level was 9.5 ng/mL)	6.1 ng/mL	Recovered
USA/2023/(Michael et al., 2023)	M	79	Liver transplant	NR	NR	5 days	NR	Weakness, diarrhea, nausea with retching, mildly tachypneic, dry cough, slight anemia, hyponatremia, acute kidney injury	26.6 ng/mL	No	NR	NR	NR	NR
USA/2022/(Sindelar et al., 2023)	F	67	Orthotopic heart transplant	3 mg AM and 2 mg PM	NR	4 days	No adjustment	Slowed speech, fatigue, weakness, loss of appetite, acute kidney injury	176.4 ng/mL	Oral phenytoin: Day 2–5, 150 mg twice, 7 doses	Hold Tac and azathioprine	NR	6.1 ng/mL	Recovered
USA/2022/(Cadley et al., 2022)	F	55	Kidney transplant	1 mg AM and 2 mg PM	NR	3 days (6 doses)	No adjustment	Acute hypoxic respiratory failure, altered mental status, and diarrhea, acute kidney injury, toxic metabolic encephalopathy	>60 ng/mL	Intravenous phenytoin: Day 3, 300 mg; Day 4, 200 mg, 2 doses; Day 5, 200 mg	Hold Tac and mycophenolate	1 mg twice daily along after discharge (when Tac level was <10 ng/mL)	<10 ng/mL	Recovered
											Receive dexamethasone 6 mg daily and one dose of tocilizumab 600 mg			
USA/2022/(Shah et al., 2022)	M	43	Orthotopic heart transplant	NR	NR	NR	NR	Worsening cough, dyspnea, hemoptysis, hyperkalemia, normal anion-gap metabolic acidosis, acute kidney injury, stable chronic pancytopenia	>60 ng/mL	Phenytoin	Inhaled tranexamic acid	NR	12.6 ng/mL	Recovered
											Oxygen supplementation			
USA/2023/(Snee et al., 2023)	F	85	Kidney transplant	2 mg twice daily	300/100 mg twice daily	5 days	Discontinue Tac	Unconsciousness, hypertension, dehydration, acute kidney injury	189 ng/mL	IV fosphenytoin 15 mg/kg, followed by tube feeding phenytoin 100 mg thrice daily	Intravenous fluids	NR	NR	Recovered
											receive antibiotics, stress dose steroids and diltiazem			
Korea/2022/(Kwon et al., 2022)	M	65	Kidney transplant	4.5 mg twice daily	NR	3 days	Discontinue Tac on Day 4 of taking Paxlovid	Headache, nausea, abdominal pain, peripheral neuropathy, acute kidney injury	>30 ng/mL	Oral phenytoin: Day 1, 200 mg thrice daily; Day 2, 200 mg–100 mg; Day 3, 100 mg	NR	Resume Tac at 90% of the baseline daily dose (when Tac level was <5 ng/mL)	8.2 ng/mL	Recovered
USA/2022/(Rose et al., 2022)	M	40	Pancreas-kidney transplant	6 mg AM/5 mg PM	150/100 mg twice daily	2 days (4 doses)	Reduce Tac to 3 mg AM/2 mg PM	Worsening fatigue and “gnawing” back and abdominal pain, hypotension, tachypneic, acute kidney injury	≥60 ng/mL	Oral rifampin: Day 3, 600 mg; Day 4, 600 mg, 2 doses	Reduce Tac to 1 mg twice daily upon admission and hold Tac on day 2	1 mg twice daily (when Tac level was 10 ng/mL)	NR	Recovered
											Intravenous a liter of normal saline			
	F	58	Lung transplant	2.5 mg twice daily	300/100 mg twice daily	3 days (6 doses)	Resume 2.5 mg twice daily	“Gnawing” abdominal pain, enteritis, nausea and vomiting, somnolence, acute kidney injury	≥60 ng/mL	Oral rifampin: Day1, 300 mg; Day 2, 300 mg, 2 doses; Day 3, 300 mg	Hold Tac	1.5 mg twice daily (when Tac level was 6 ng/mL)	NR	Recovered
											Intravenous fluids, dexamethasone, vancomycin, and ceftriaxone			
Japan/2023/(Shiohira et al., 2024)	F	61	Lung transplant	1 mg twice daily	150/100 mg twice daily	3 days (6 doses)	Discontinue Tac	Vomit, diarrhea, acute kidney injury	>60 ng/mL	NR	NR	1 mg daily (when Tac level was 2.1 ng/mL)	6.2 ng/mL	Recovered
USA/2022/(Lindauer and Hamel, 2022)	F	41	Kidney transplant	4 mg daily	NR	2.5 days (5 doses)	No adjustment	Hyperkalemia, acute kidney injury	>60 ng/mL	NR	NR	NR	NR	Recovered
USA/2022/(Mecadon et al., 2022)	M	40	Kidney transplant	1 mg AM and 2 mg PM	NR	5 days	Discontinue Tac	NR	25.1 ng/mL	NR	NR	1 mg AM and 2 mg PM	17 ng/mL	Recovered
	F	41		6 mg AM and 7 mg PM	NR	5 days	Discontinue Tac	NR	21.7 ng/mL	NR	NR	6 mg AM and 7 mg PM	14 ng/mL	Recovered
	M	45		1 mg AM and 2 mg PM	NR	5 days	Discontinue Tac	NR	5.1 ng/mL	NR	NR	1 mg AM and 2 mg PM	25.9 ng/mL	Recovered
	M	77		1 mg twice daily	NR	5 days	No adjustment	Weakness, confusion, acute kidney injury	44.5 ng/mL	NR	Hold Tac	NR	5 ng/mL	Recovered
France/2022/(Guyon et al., 2022)	F	58	Liver transplant	2 mg daily	150/100 mg twice daily	5 days	Discontinue Tac	Arthralgia, asthenia, and diarrhea, acute kidney injury, hyperkaliemia, metabolic acidosis	111 ng/mL	NR	Dialysis	NR	NR	Recovered
China/2023/(Chen et al., 2023)	F	39	Myasthenia gravis	6 mg daily	300/100 mg twice daily	5 days	Discontinue Tac on the second day of Paxlovid use	Mild elevation of liver enzymes	>30 ng/mL	NR	NR	Reintroduce at 1 mg q12h, gradually increase to 2 mg q12h, and change to 1 mg q12h due to Wuzhi capsule	2.5–4.6 ng/mL	Recovered
China/2023/(Luo et al., 2023)	M	56	Kidney transplant	2 mg twice daily	300/100 mg twice daily	4 days	Reduce Tac to 1 mg twice daily	Diarrhea, dehydration, a drowsy state, tachypnea, tachycardia, metabolic acidosis, acute kidney injury, diabetic ketoacidosis, a hyperglycemic hyperosmolar state	NR	No	Stop diarrhea	0.5 mg twice daily	4.56 ng/mL	Recovered
											Restore intravascular volume			
											Correct electrolyte abnormalities, acidosis and hyperglycemia			
China Taiwan/2023/(Chiu et al., 2023)	F	33	Small bowel transplant	3 mg daily	300/100 mg twice daily	12 h	No adjustment	One episode of bloody stool and dizziness with a fainting sensation	NR	No	Hold all drugs	3 mg daily	2.8 ng/mL	Recovered
											Re-start immunosuppressants at the next day			
USA/2023/(Maynard et al., 2023)	F	69	Liver transplant	1 mg twice daily	NR	NR	NR	Headache, weakness, nausea, epigastric abdominal pain, elevated alkaline phosphatase	>40.0 ng/mL	NR	Hold Tac	NR	NR	Recovered
											Received supplemental O2			
Japan/2023/(Tomida et al., 2023)	M	50	Kidney transplant	2.5 mg daily	150/100 mg twice daily	5 days	No adjustment	Abdominal pain with paralytic ileus, acute kidney injury, hyperkalemia	96.4 ng/mL	No	Hold Tac	1.5 mg daily (when Tac level was 5.5 ng/mL)	4.0 ng/mL	Recovered
											Fast and fluid supplementation			
Japan/2023/(Tsuzawa et al., 2023)	M	Forties	Lung transplant	0.5 mg twice daily	300/100 mg twice daily	5 days	Discontinue Tac during NMV/r, and restart Tac 1.0 mg daily after completion of NMV/r	Increased serum creatinine	31.6 ng/mL	NR	Hold Tac	0.5 mg twice daily (when Tac level was <16 ng/mL)	9.6 ng/mL	Recovered
Spain/2023/(Cordero and de Vicente, 2023)	F	83	Kidney transplant	5 mg daily	2 tablets every 12 h (dose adjusted to renal function)	3 days	No adjustment	An impaired level of consciousness, amnesia, tremors, decreased intake, mucocutaneous dryness, acute kidney injury	112 ng/mL	NR	Hold Tac	Reintroduce at 1 mg (when Tac levels was 8.2 ng/mL), and gradually increase to 3 mg daily	5.7–6.2 ng/mL	Recovered
											Treated with 1,500 mL of sodium chloride 0.9% saline solution daily			
China/2023/(Xiong et al., 2023)	F	57	Lung transplant	2.5 mg twice daily	NR	NR	Hold Tac	Vomiting, fatigue, headaches, myalgia	>60 ng/mL	No	Hold Tac	1.5 mg daily	9.6 ng/mL	NR
USA/2023/(Zaarur et al., 2023)	F	13	Ulcerative colitis	5 mg twice daily	150/100 mg twice daily	3.5 days (7 doses)	No adjustment	Chest pain, hyperkalemia, acute kidney injury	121.2 ng/mL	Rifampicin: 600 mg once daily for 3 days	Discontinue Tac	NR	2.9 ng/mL	NR
Japan/2024/(Akamatsu et al., 2024)	M	60	Interstitial pneumonia associated with dermatomyositis	1.5 mg twice daily	NR	NR	Hold Tac	Vomiting, fatigue, headaches, myalgia	>60 ng/mL	No	Hold Tac	2 mg daily (when Tac levels was 1.9 ng/mL)	NR	Recovered
											NMV/r adjusted to remdesivir			
China Taiwan/2024/(Lo et al., 2024)	F	64	Lung transplant	4 mg twice daily	300/100 mg twice daily	5 days	No adjustment	Weakness, fatigue, hyponatremia	>60 ng/mL	No	Hold Tac and mycophenolate mofetil	2 mg twice daily	NR	Recovered
Japan/2024/(Yamamoto et al., 2024)	M	43	Systemic Lupus Erythematosus	3 mg daily	300/100 mg twice daily	2 days	Reduced Tac to 2 mg daily	Severe headache, nausea, vomiting, hypomagnesemia	77.1 ng/mL	No	Hold Tac	NR	NR	NR
China/2024/(Zhang et al., 2024)	F	37	Systemic Lupus Erythematosus	1 mg twice daily	300/100 mg twice daily	5 days	No adjustment	Persistent abdominal pain, nausea, vomiting, paralytic ileus, slight hypocomplementemia	>30 ng/mL	No	Hold Tac	1 mg twice daily (when Tac levels was 4.4 ng/mL)	4.4 ng/mL	Recovered
											Diet ban, gastrointestinal decompression, enema, and parenteral feeding			
China/2024/(Shen et al., 2024)	M	32	Kidney transplant	4 mg twice daily	150/100 mg twice daily	5 days	Discontinue Tac	Fatigue, serum creatinine increased	72.8 ng/mL	No	NR	0.5 mg twice daily (when Tac levels was 4.06 ng/mL)	4.06 ng/mL	Recovered
	M	66	Hematopoietic stem cell transplant	2 mg twice daily	300/100 mg twice daily	5 days	Reduced Tac to 1.5 mg twice daily	Fatigue, nauseous, increased serum creatinine	>40 ng/mL	No	Hold Tac	Increase from 0.5 mg twice daily to 1.5 mg twice daily	10.07 ng/mL	Recovered
											Reduce isavuconazole sulfate from 200 mg to 100 mg four times daily for days 5–8			
USA/2024/(Marsh et al., 2024)	F	75	Heart transplant	NR	300/100 mg twice daily	2.5 days (5 doses)	Discontinue Tac	Headache, malaise, acute kidney injury	>60 ng/mL	Oral phenytoin: 200 mg twice daily, 5 doses	NMV/r adjusted to remdesivir	Resume at a 40% dose reduction from baseline dose	NR	NR
	M	43	Kidney transplant	NR	150/100 mg twice daily	2 days (4 doses)	No adjustment	Worsened fatigue, decreased urination, nausea, acute kidney injury	>60 ng/mL	Oral phenytoin: 100 mg thrice daily, 4 doses	Hold Tac	Previous dose (when Tac levels was <1.0 ng/mL)	9.6 ng/mL	NR
	M	60	Lung transplant	NR	300/100 mg twice daily	1 day (2 doses)	No adjustment	Sinus congestion, dysphonia, and oxygen desaturations to 88% with walking but with quick recovery	>60 ng/mL	Oral phenytoin: 100 mg thrice daily for 5 days	Discontinue Tac	NR	5.8 ng/mL	NR
											Receive a dose of bebtelovimab			
	M	53	Kidney transplant	NR	300/100 mg twice daily	1 day (2 doses)	No adjustment	Intractable nausea, vomiting, elevated aspartate transaminase and creatinine levels	>60 ng/mL	Oral phenytoin: 100 mg twice daily, 4 doses	Hold Tac	Resume at a lower dose	9.2 ng/mL	NR
											Receive a dose of bebtelovimab			
	M	57	Heart transplant	NR	NR	3 days (7 doses)	No adjustment	Severe headache	59.9 ng/mL	Oral phenytoin: 200 mg twice daily, 3 doses, followed by 100 mg twice daily, 2 doses	Hold Tac	Resume (when Tac levels was 11.5 ng/mL)	5.6 ng/mL	NR
											NMV/r adjusted to remdesivir			

Tac, tacrolimus; NMV/r, nirmatrelvir/ritonavir; CYP, cytochrome P450; NR, not reported.

The reported toxicity manifestations were diverse, encompassing nephrotoxicity, gastrointestinal toxicity, neurotoxicity, metabolic disturbances, cardiovascular toxicity, hematological toxicity, musculoskeletal toxicity, and hepatotoxicity. These toxic effects generally manifested during NMV/r treatment and could persist post-treatment.

Treatment regimens for acute Tac toxicities varied. Of the 38 patients, 17 received the full prescribed dose of NMV/r, while 10 had their NMV/r doses adjusted based on renal function. The dosing details for the remaining 11 patients were not explicitly reported. Tac management during NMV/r treatment also differed: 20 patients maintained their original dosage, 5 opted for reduced doses, 8 discontinued Tac immediately, 1 stopped Tac on the 4th day post-NMV/r, and another stopped on the second day post-NMV/r. In 3 cases, specific management strategies were not documented.

Observed Tac concentrations varied significantly, with the maximum recorded level reaching 189 ng/mL. The minimum concentration associated with toxic symptoms was 24.3 ng/mL, surpassing the therapeutic levels. Of the 38 patients, only 14 patients were treated with CYP inducers, including 10 with phenytoin at doses ranging from 200 mg/d to 600 mg/d for 2–5 days, and 4 with rifampin at doses ranging from 300 mg/d to 600 mg/d for 2–3 days. Following hospitalization and treatment, 26 patients resumed Tac therapy: 8 patients reverted to their initial dose, 17 proceeded with a reduced dose, and in one case, the details were not specified. Notably, 14 of these patients resumed Tac therapy at concentrations ≤10 ng/mL and 3 patients at concentrations ≤16 ng/mL. 29 patients showed clear improvement in symptoms, while the discharge status of 9 patients was not mentioned.

## 4 Discussion

Tac, a calcineurin inhibitor, is widely utilized for immunosuppression in solid organ transplant recipients. It has garnered attention for its efficacy in treating SLE, leading to expanded usage in this context (Watanabe et al., 2016). Despite its effectiveness in managing SLE, Tac poses challenges due to its narrow therapeutic window, typically targeting concentrations of 4–6 ng/mL in SLE patients (Fanouriakis et al., 2019). Previous investigations have suggested that patients with various immune-mediated diseases commonly experienced varying degrees of elevation in Tac levels during or following NMV/r use, potentially leading to acute toxicity. The primary toxicities observed in current cases were nephrotoxicity and neurotoxicity. Acute Tac-induced nephrotoxicity, characterized by a moderate increase in serum creatine levels due to acute afferent arteriolar vasoconstriction, primarily affects tubular epithelial cells, vascular endothelial cells, arteriolar myocytes, and interstitial fibroblasts. These cellular damages result from high concentrations of Tac-binding proteins that inhibit calcineurin activity, leading to functional and structural renal impairment (Braithwaite et al., 2021). Unlike nephrotoxicity, Tac-induced neurotoxicity, typically diagnosed based on neurological symptoms and severity, may stem from inhibited calcineurin activity, essential for neurological function (Bechstein, 2000). Neurotoxicity is also associated with posterior reversible encephalopathy syndrome (PRES), often triggered by hypertension, sepsis, or renal failure, characterized by vasogenic edema due to blood-brain barrier dysregulation and impaired cerebral vasoconstriction (Dhar, 2017; Farouk and Rein, 2020). The incidence and severity of acute nephrotoxicity and neurotoxicity correlate with supratherapeutic Tac trough concentrations, (Sikma et al., 2018; Miano et al., 2020), often observed in transplant patients with levels exceeding 15 ng/mL (Braithwaite et al., 2021). However, the precise threshold for acute toxicities in SLE patients remains unclear.

Tac, primarily metabolized by CYP3A4 and serving as a substrate for P-glycoprotein, undergoes first-pass intestinal metabolism, with an intestinal availability of 0.14 and a hepatic availability of 0.96 (Tomida et al., 2023). Ritonavir inhibits P-glycoprotein, thereby diminishing Tac absorption and subsequently elevating Tac levels. Moreover, ritonavir tightly binds to the active site of CYP3A4, forming an irreversible bond with the heme iron via the thiazole nitrogen. This action decreases the redox potential of the CYP protein and impedes its reduction by CYP450 reductase (Sevrioukova and Poulos, 2010). Consequently, ritonavir can disrupt Tac metabolism by irreversibly inhibiting CYP3A4 and P-glycoprotein activity, resulting in increased systemic exposure and reduced metabolic clearance of Tac (Fishbane et al., 2022). A pharmacokinetic evaluation revealed that co-administration with NMV/r resulted in an 18.7-fold increase in Tac bioavailability and a 35% reduction in clearance (Tomida et al., 2023). In healthy volunteers, steady-state concentrations of ritonavir (100 mg/day) led to a 17-fold and 57-fold elevation in the Tac concentration at 24 h (C_24_) and the area under the plasma concentration-time curve (AUC_0-inf_), respectively, with Tac half-life extending from 32 h to 232 h (Badri et al., 2015). Another prospective pharmacokinetic study demonstrated that ritonavir could sustain elevated Tac levels even after discontinuation of NMV/r, possibly due to continued inhibition of CYP3A metabolism (Xu et al., 2024). The half-life of Tac is approximately 35 h, the metabolic inhibition of Tac persisted up to 110 h after ritonavir discontinuation (Yamamoto et al., 2024). In our case, despite Tac resumption following NMV/r cessation, a significant surge in Tac levels, accompanied by nephrotoxicity and neurotoxicity, was observed in the patient. This underscores the sustained inhibitory effect of NMV/r on Tac, persisting even after discontinuation.

Currently, treatment of Tac toxicity relies mainly on supportive care, as there is no specific antidote available. Ceschi et al. proposed early interventions such as gastrointestinal decontamination using activated charcoal or nasogastric aspiration to potentially reduce Tac absorption in cases of acute overdose (Ceschi et al., 2013). However, the efficacy of these measures is limited by Tac’s high protein binding and minimal biliary excretion (Quirós-Tejeira et al., 2005; Jantz et al., 2013). Due to Tac’s lipophilic nature and extensive erythrocyte binding (99%), extracorporeal removal is ineffective in Tac toxicity cases. Nonetheless, renal replacement therapy may be necessary to manage volume overload or electrolyte disturbances in cases of acute Tac-induced nephrotoxicity (Naccarato et al., 2021). Given the close relationship between Tac toxicity and enzyme inhibition, inducing Tac metabolism via CYP activation is considered a potential therapeutic approach. Literature suggests that the half-life of the CYP3A4 enzyme is approximately 2–3 days, with full recovery of enzyme activity requiring several days for the regeneration of new enzymes (Chen and Raymond, 2006; Magnusson et al., 2008). CYP3A4 inducers, such as phenytoin or rifampin, offer alternative therapeutic approaches to enhance CYP3A4 enzyme activity. Phenytoin, in particular, presents potential advantages in managing severe neurological symptoms induced by Tac toxicity (e.g., seizures or convulsions) (Hoppe et al., 2022). Hence, phenytoin was administered in our case to decrease Tac blood concentration. Phenytoin exerts a robust induction effect on CYP3A by activating the constitutive androstane receptor (CAR), which binds to the promoter region of CYP3A genes. Moreover, the induction effect of phenytoin on enzymes is dose-dependent (Brodie et al., 2013). However, phenytoin’s narrow therapeutic window and relatively long half-life (approximately 42 h orally) may affect the metabolism of other drugs due to its hepatic enzyme induction, increasing the risk of adverse reactions (Xiong et al., 2023). Therefore, it is necessary to monitor the blood concentration of phenytoin. Detailed pharmacokinetic data on the interaction between phenytoin and Tac in SLE patients is lacking. A reported case noted that the elimination half-life of Tac was <85.5 h within 1–6 days after discontinuation of NMV/r and Tac (Shiohira et al., 2024). In our case, the patient had discontinued NMV/r 4 days before admission and was concurrently administered phenytoin for 5 days upon admission, resulting in a Tac elimination half-life of 55.87 h. However, due to differences in medication regimens between the two cases, a direct comparison of Tac elimination half-lives is not feasible. Larger-scale studies are required to determine whether phenytoin can shorten Tac’s elimination half-life. Once Tac concentrations approach the therapeutic target, discontinuation of phenytoin is recommended (Lange et al., 2017).

Given the narrow therapeutic range of Tac and its significant drug-drug interactions (DDIs) with NMV/r, leading to serious toxicity, effective management strategies are essential. However, current literature on managing this DDI, especially in organ transplant recipients, is limited to a few small-scale retrospective studies. For patients on NMV/r, strategies such as temporarily holding or reducing Tac dosage and closely monitoring Tac levels may help prevent toxicity. The French Society of Pharmacology and Therapeutics recommends suspending Tac 12 h before starting NMV/r and resuming the usual daily dose (DD) 24 h after the last NMV/r dose (Lemaitre et al., 2022). Devresse et al. outlined a protocol involving discontinuing Tac 12 h before NMV/r initiation and administrating 20% of the cyclosporine dose. Ten of these patients resumed Tac on the second day after NMV/r discontinuation (Devresse et al., 2022). A similar approach was suggested by Lange et al. (2022) Salerno et al. shared their experiences with 25 solid organ transplant recipients, adjusting the regimen by either withholding Tac/mTOR inhibitors or reducing the cyclosporine dose to 20% of the baseline daily dose during NMV/r treatment, and restarting Tac 2–5 days post-treatment (Salerno et al., 2022). Dewey et al. described 12 lung transplant recipients who started NMV/r 10–14 h after the last Tac dose, with most reintroducing Tac within 4 days post-NMV/r (Dewey et al., 2023). Another retrospective study recommended withholding Tac for 24 h and resuming it 72 h after the last NMV/r dose (Giguère et al., 2023). Despite noting supratherapeutic Tac levels post-NMV/r, these studies reported minimal severe complications, suggesting the efficacy and safety of a well-timed NMV/r initiation and Tac resumption strategy in long-term Tac patients.

Estimating the overall change in Tac exposure poses a challenge, underscoring the importance of measuring Tac concentrations on days 3, 6, and 7 following NMV/r initiation to inform Tac reintroduction (Wang et al., 2022). A retrospective study, acknowledging the variance in Tac formulations, proposed differing reintroduction timelines: 24 h after the last Paxlovid dose for immediate-release Tac, and 48 h for sustained-release or long-acting formulations (Belden et al., 2023). These findings indicate that NMV/r does not necessarily preclude Tac usage, and standardized management can be mitigate toxicity arising from drug interactions. However, the majority of evidence for Tac dose adjustment arises from organ transplant cases, with a noticeable dearth of data for SLE. At our institution, clinical pharmacists play a vital role in transplant and emergency pharmacotherapy. They collaborate with clinical physicians to optimize drug treatment management for patients with acute toxicity. Unfortunately, in this instance, the patient self-administered NMV/r without physician or pharmacist consultation. Therefore, healthcare providers should prioritize vigilance toward potential drug interactions when prescribing medications, while patients should be educated about such interactions before initiating medication. Additionally, for Tac users, alternative COVID-19 antivirals with fewer interactions like oral molnupiravir or intravenous remdesivir, should be considered. However, the parenteral nature of remdesivir may limit its practically for outpatient administration.

## 5 Conclusion

This case underscores the critical importance of remaining vigilant regarding the drug-drug interactions (DDIs) between Tac and NMV/r in SLE patients, emphasizing the persistent nature of enzyme inhibition. It also highlights the indispensable role of therapeutic drug monitoring in Tac therapy management. Furthermore, the efficacy and safety of phenytoin as a pharmacokinetic inducer in mitigating Tac toxicity are underscored by this case. These findings underscore the complexity of managing DDIs in Tac recipients and emphasize the essential involvement of pharmacists in clinical decision-making and close monitoring in such scenarios.

## 6 The patient’s perspective

We reported the following about the patient’s experience: “I am a long-term SLE patients who has been taking Tac. This experience has made me deeply aware of the dangers of drug-drug interactions. During this visit and hospitalization, I received comprehensive treatment from hospital doctors, pharmacists, and nurses. After the treatment, my swallowing function and kidney function have significantly improved. Although I experienced symptoms of increased heart rate, I am satisfied with the treatment outcomes.”

## Data Availability

The original contributions presented in the study are included in the article/supplementary material, further inquiries can be directed to the corresponding authors.
